# A statistical, voxelised model of prostate cancer for biologically optimised radiotherapy

**DOI:** 10.1016/j.phro.2022.02.011

**Published:** 2022-03-06

**Authors:** Robert N Finnegan, Hayley M Reynolds, Martin A Ebert, Yu Sun, Lois Holloway, Jonathan R Sykes, Jason Dowling, Catherine Mitchell, Scott G Williams, Declan G Murphy, Annette Haworth

**Affiliations:** a Institute of Medical Physics, School of Physics, University of Sydney, Sydney, New South Wales, Australia; b Liverpool Cancer Therapy Centre, South Western Sydney Local Health District, Liverpool, New South Wales, Australia; c InghamInstitute for Applied Medical Research, Liverpool, New South Wales, Australia; d Auckland Bioengineering Institute, University of Auckland, New Zealand; e Department of Radiation Oncology, Sir Charles Gairdner Hospital, Nedlands, Western Australia, Australia; f School of Physics, Mathematics and Computing, University of Western Australia, Crawley, Western Australia, Australia; g 5D Clinics, Claremont, Western Australia, Australia; h Centre for Medical Radiation Physics, University of Wollongong, Wollongong, New South Wales, Australia; i South Western Sydney Clinical School, University of New South Wales, Sydney, New South Wales, Australia; j Department of Radiation Oncology, Sydney West Radiation Oncology Network, Blacktown Cancer & Haematology Centre, Blacktown, New South Wales, Australia; k Department of Radiation Oncology, Sydney West Radiation Oncology Network, Crown Princess Mary Cancer Centre, Westmead, New South Wales, Australia; l School of Mathematical and Physical Sciences, University of Newcastle, Newcastle, New South Wales, Australia; m CSIRO Health and Biosecurity, The Australian e-Health and Research Centre, Herston, Queensland, Australia; n Department of Pathology, Peter MacCallum Cancer Centre, Melbourne, Victoria, Australia; o Sir Peter MacCallum Department of Oncology, University of Melbourne, Melbourne, Victoria, Australia; p Division of Radiation Oncology and Cancer Imaging, Peter MacCallum Cancer Centre, Melbourne, Victoria, Australia; q Division of Cancer Surgery, Peter MacCallum Cancer Centre, Melbourne, Victoria, Australia

**Keywords:** Prostate cancer, Tumor biology, Statistical atlas, Radiobiology, Biological atlas

## Abstract

**Background and purpose:**

Radiation therapy (RT) is commonly indicated for treatment of prostate cancer (PC). Biologicallyoptimised RT for PC may improve disease-free survival. This requires accurate spatial localisation and characterisation of tumour lesions. We aimed to generate a statistical, voxelised biological model to complement *in vivo*multiparametric MRI data to facilitate biologically-optimised RT.

**Material and methods:**

Ex vivo prostate MRI and histopathological imaging were acquired for 63 PC patients. These data were co-registered to derive three-dimensional distributions of graded tumour lesions and cell density. Novel registration processes were used to map these data to a common reference geometry. Voxelised statistical models of tumour probability and cell density were generated to create the PC biological atlas. Cell density models were analysed using the Kullback–Leibler divergence to compare normal vs. lognormal approximations to empirical data.

**Results:**

A reference geometry was constructed using ex vivo MRI space, patient data were deformably registered using a novel anatomy-guided process. Substructure correspondence was maintained using peripheral zone definitions to address spatial variability in prostate anatomy between patients. Three distinct approaches to interpolation were designed to map contours, tumour annotations and cell density maps from histology into ex vivo MRI space. Analysis suggests a log-normal model provides a more consistent representation of cell density when compared to a linear-normal model.

**Conclusion:**

A biological model has been created that combines spatial distributions of tumour characteristics from a population into three-dimensional, voxelised, statistical models. This tool will be used to aid the development of biologically-optimised RT for PC patients.

## Graphical abstract

**Figure:**
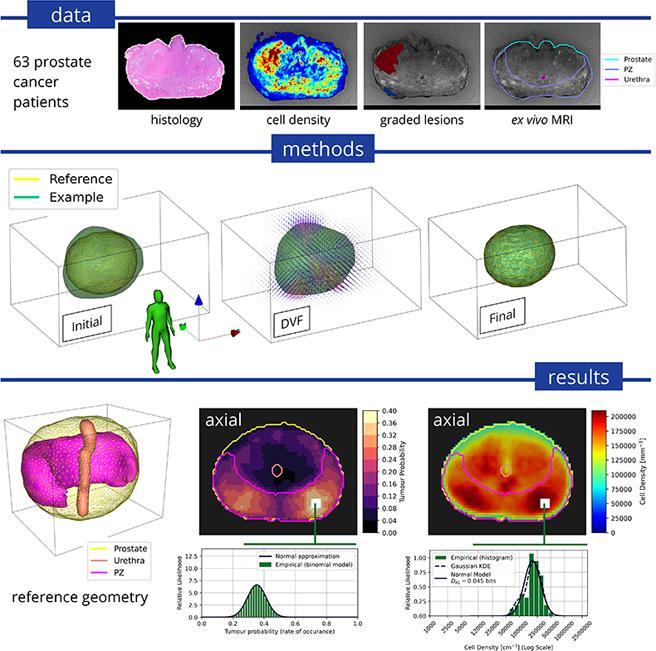

## 1 Introduction

Prostate cancer (PC) is the most common cancer diagnosis in men worldwide, and a leading cause of cancer-related death [1]. Patients with organ-confined disease are generally considered for active surveillance programs or radical treatment with curative intent, such as prostatectomy (surgical removal of the whole prostate) and radiation therapy (RT) [2]. Current RT standards of care aim to deliver a uniform radiation dose to the entire prostate [3]. Although RT is effective in dramatically reducing cancer-related death for the 60% of PC patients for whom this treatment is indicated [4], around one in four patients receiving RT will relapse within 5 years [5]. Local PC recurrence typically occurs at tumour foci, which can be identified on multi-parametric magnetic resonance imaging (mpMRI) [6]. Although increasing the radiation dose to the entire prostate volume would likely improve rates of tumour control, this is not possible with existing technology due to risks of exceeding tolerance doses to nearby healthy tissue. Considering this, a potential approach to improve RT efficacy is to deliver higher boost doses of radiation to small subvolumes of the prostate identified as cancerous [7], [8], [9], [10], typically using mpMRI to define these subvolumes [11], [12], [13], [14], [15]. A number of clinical trials have investigated this MRI-based focal boost RT [16], [5], [17], [18], with at least one modern study reporting improvements in disease-free survival [19].

Whilst MRI is now widely accepted as a superior imaging modality for defining pelvic anatomy and focal subvolumes for PC RT, the use of mpMRI for tumour characterisation for biologically-optimised radiotherapy approaches is still in its infancy [20], [21]. Although this concept was proposed more than twenty years ago [22], clinical translation has been hampered by the lack of high-resolution imaging data to accurately define tumour biology. Modern imaging techniques, in combination with artificial intelligence (AI) methods, now provide a framework for producing spatial maps of tumour biology [23], [24], [25], [26]. Validation of computational models to predict biological characteristics using patient imaging data, however, remains challenging. Ground truth is difficult to ascertain; histopathological analysis of surgically removed prostates is considered the gold standard, but accurate correlation of histology and mpMRI is hampered by significant differences in features between these modalities and physical deformation of the prostate following removal [27], [28], [29], [30]. We previously reported a novel approach, using *ex vivo* MRI, to minimise uncertainties during co-registration, however, quantifying the accuracy of this process is difficult [31]. Consequently, such models will always carry a degree of uncertainty in predicting spatial distributions of biological characteristics.

To address issues of uncertainty in the application of predictive models, a population-based statistical model of PC biology was developed to be used as a probabilistic prior to improve the accuracy and robustness of predictions based on patient imaging data alone. Additionally, this model represents a new research framework to explore biologically-optimised RT paradigms by encoding uncertainty in biological parameters at a voxel level. The aims for this study were to design and implement a framework to derive a statistical, voxelised model of PC tumour biology.

## 2 Material and Methods

### 2.1 Study Data

Seventy PC patients scheduled for radical prostatectomy were recruited to a Human Research Ethics Committee approved project (HREC15/PMCC125) [31]. Of these, 63 had complete imaging and histology data necessary for this specific project. Patient details and study inclusion criteria are available in supplementary material Section 1. Prior to prostatectomy, each patient underwent *in vivo* mpMRI as per PI-RADS and ESUR guidelines [32]. These *in vivo* data were not included in the development of this model, hence no further details are provided here. Following resection, each prostate was set in agarose gel in a specially designed sectioning box which had cutting slots at 5 mm intervals. The specimen contained within the sectioning box underwent *ex vivo* imaging. The *ex vivo* imaging data were acquired with axial T2w images, with an in-plane resolution of 0.22 mm and slice thickness 2.5 mm (further details in supplementary material Section 2). The sectioning and preparation of whole mount histological specimens were described in detail by Reynolds et al. [31]. In brief, prostate specimens were sectioned in 5 mm increments using the cutting slots in the sectioning box, after which 3 μm thick sections were microtomed from the top surface of each block, mounted on 25×75 mm slides and stained with haematoxylin and eosin. The sections at the apex and base were cut parasagittally according to pathology protocols and excluded from this process. An expert urological pathologist annotated and assigned a Gleason Score to each lesion.

### 2.2 Data pre-processing

Cell density maps were computed using an established method [33] on high-resolution images of each slide obtained at 20× magnification. To ensure cell density maps could be compared between patients, stain normalisation was first performed to account for differences in staining intensities using Reinhard’s method [34].

**Fig. 1:**
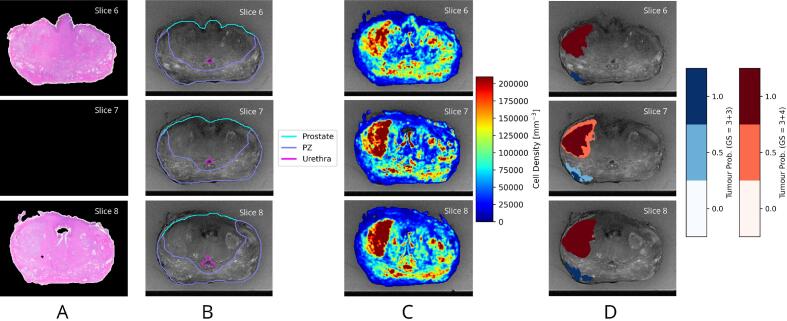
Illustration of the interpolation processes used to generate data at MRI slice locations corresponding to gaps between adjacent histology slides (i.e. slice 7, as shown in A). (B) Prostate, peripheral zone (PZ) and urethra delineations are defined using morphological contour interpolation. (C) The cell density is defined using linear interpolation. (D) The graded tumour lesions are interpolated with probabilistic weightings for each individual Gleason score (GS). Although tumour lesions with different Gleason grades are shown overlaid on the same axial slices, these probabilistic labels are represented as separate images to handle cases where adjacent slices have different Gleason grades. In such cases, a single voxel could have a weighting of 0.5 for each Gleason grade.

Each patient’s set of digitised histology slides was co-registered with its corresponding *ex vivo* axial T2w MR image using a previously developed registration pipeline [31]. Prostate volumes were contoured on the *ex vivo* MRI by an experienced radiation oncologist for the first 39 patients, after which a deep learning model was trained using these data to automatically contour the remaining patients, with manual editing performed by a radiotherapy imaging scientist. Further details of the deep learning model are available in supplementary material Section 3. The peripheral zone (PZ) and urethra were contoured manually on the registered histology images by radiotherapy imaging scientists (Fig. 1). Contouring was performed using 3D Slicer [35] (www.slicer.org/).

Whole-mount pathology sections represent tissue at 5 mm intervals and so data were missing for every second axial MRI slice (acquired with 2.5 mm thick slices). A framework was developed to interpolate these data using the adjacent slices. Different approaches were used for contours, cell density maps, and tumour annotations. Contours were defined using morphological interpolation [36], which was designed to create a smooth change in shape of anatomical regions. Cell density maps were linearly interpolated using adjacent slices, which ensured interpolated values were within the range of actual values on adjacent slices. Following this, the cross-sectional cell density was converted to volumetric cell density by raising values to the power of 3/2. For tumour annotations a probabilistic approach was used: a separate 3D image was created to represent the label corresponding to lesions with each Gleason score. On axial slices corresponding to co-registered histology slides the lesion labels were assigned a value of 1, and for the slices immediately before and after the label was extended with a weighting of 0.5. Therefore, at locations where adjacent histology slides had lesions of the same grade, the probabilistic lesion label on the missing MRI slice was assigned a value of 1. These processes are illustrated in Fig. 1.

Following interpolation, all co-registered data, comprising axial T2w MRI, contours, cell density, and probabilistic graded tumour annotations, were resampled to an isotropic 0.8×0.8×0.8 mm^3^ resolution to match that of *in vivo* T2w MRI data.

### 2.3 Generation of reference geometry

**Fig. 2:**
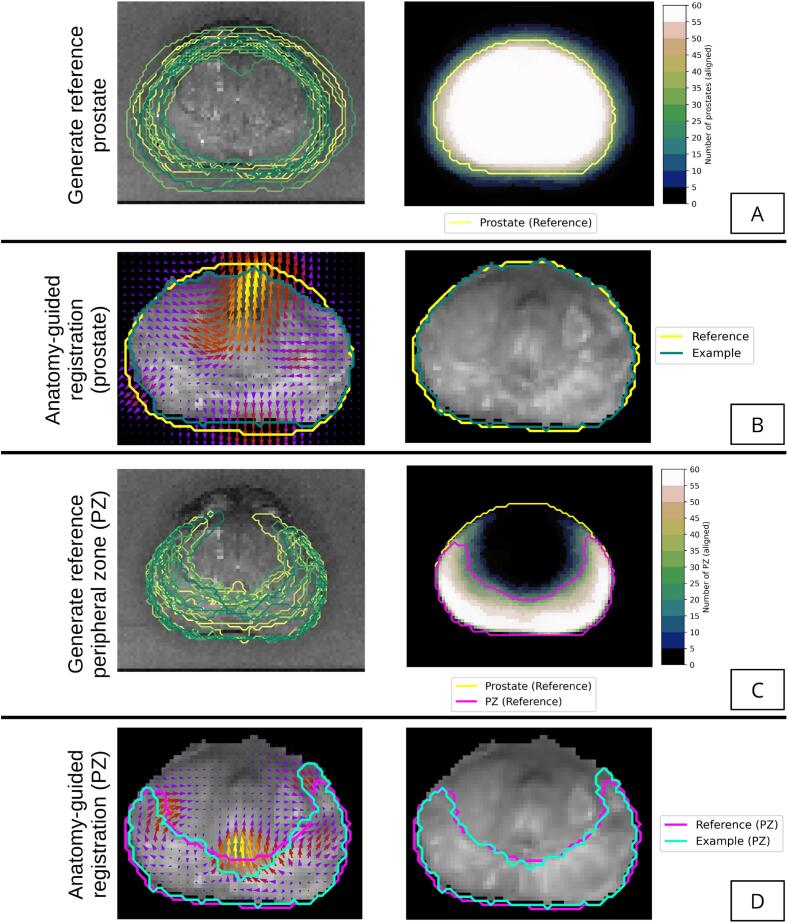
The registration and model geometry framework was designed to ensure anatomical correspondence was maintained as data was aligned to the reference space, and removed bias by using data from all patients to define the reference geometry. (A) Aligned prostate contours were used to define the reference geometry for the whole prostate based on overlap of at least half the patient contours. (B) Illustration of the structure-guided registration process with distance-preserving regularisation applied to the whole prostate contour of one patient (left) and resulting deformed MRI (right). (C) Aligned peripheral zone (PZ) contours are used to define the reference geometry for the PZ based on overlap half the patient contours. (D) Illustration of the structure-guided registration process with distance-preserving regularisation applied to the PZ contour of one patient (left) and resulting deformed MRI (right).

A “reference prostate” was generated using information from all 63 patients. First, a three-dimensional Cartesian reference space was created, defining three orthogonal axes. Prostate contours from *ex vivo* MRI space were aligned to this space by translating the prostate volume centroid to the origin and then rotating to align the three principal moments to the reference axes. This rotation was initialised using the imaging axes and then refined using the principal moments, which ensured no axes would be flipped. The consensus volume was calculated as the overlap of half or more of these 63 aligned volumes, and this defined the reference prostate (Fig. 2A) which was used as a fixed template to which all patient data were registered.

### 2.4 Registration to the reference geometry

The primary goal of the registration of patient data to the reference prostate was to maintain anatomical correspondence, as the patient imaging space was warped to match the reference.

**Fig. 3:**
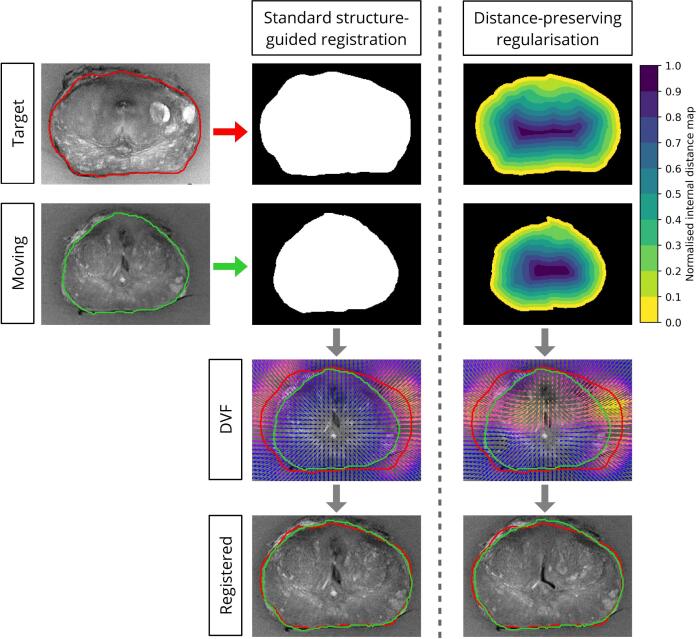
Illustration of the novel deformable registration process using distance-preserving regularisation, compared with standard structure-guided registration. A normalised distance map was generated, which was used to drive a log-domain, symmetric-forces demons-based deformable image registration process. While both methods accurately matched volume boundaries (bottom row), the new method is mathematically guaranteed to preserve the relative distance from any point to the volume boundary.

An anisotropic similarity transform was computed to scale and rigidly align (translate and rotate) the entire prostate volume to the reference. Next, a novel deformable registration technique using distance-preserving regularisation was developed to refine this co-registration. This approach employed a log-domain, symmetric-forces demons-based algorithm to register the normalised distance maps of patient and reference prostate volumes (Fig. 3). This process is an extension to standard structure-guided registration techniques, which are used to accurately align the boundaries of two volumes but do not reliably characterise internal deformations [37]. See supplementary material Section 4 for further details.

The result of this first registration step was a set of 63 independently co-registered prostate volumes (Fig. 2B). The PZ contours from the co-registered patient data were combined to define a reference PZ using a similar process that generated the reference prostate (Fig. 2C), and another registration stage was used to warp each PZ to the reference PZ (Fig. 2D) using the same distance-preserving regularisation. This step was critical to ensure anatomical correspondence with the prostate substructure preserved. An additional constraint forced deformation at the prostate border to zero to ensure the PZ-to-PZ registration would not distort results from the previous registration step in other regions of the prostate. A published splining technique [38] was used to generate a reference urethra volume using a predefined radius of 1.5 mm, to serve as an aid to visualisation.

**Fig. 4:**
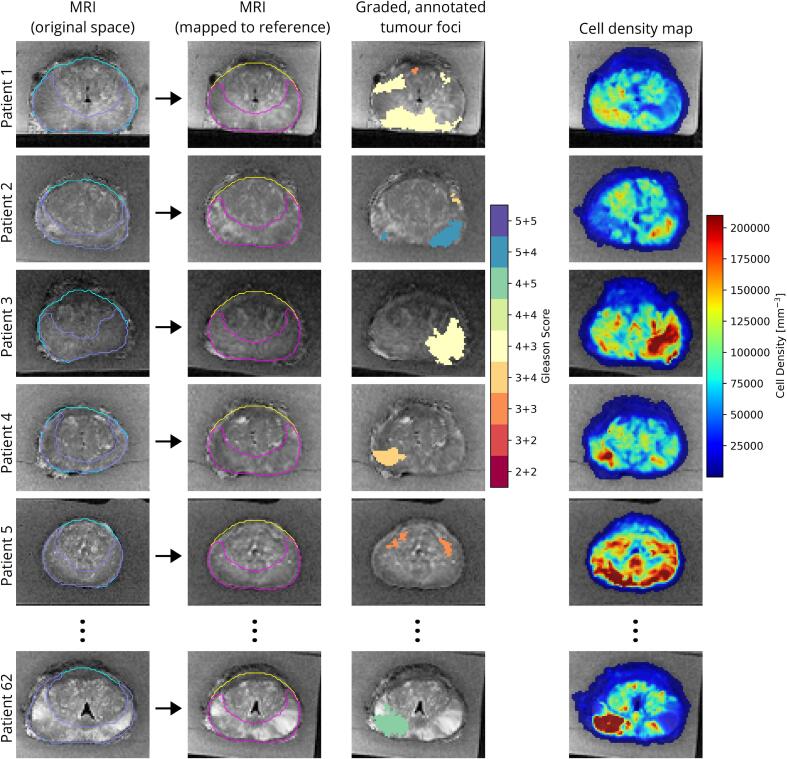
The novel co-registration process was used to map data from every patient into the reference space. This was achieved using distance-preserving deformable registration from the patient geometry (leftmost column) to the reference geometry (second to left column) to define a transformation. The graded tumour lesions (third from left column) and cell density maps (rightmost column) were mapped to the reference geometry using this derived transform.

For each individual patient the chain of transformations comprising the anisotropic similarity transform and prostate-based and PZ-based deformable transforms were used to map the annotated tumour lesions and cell density into the reference space (Fig. 4).

Image processing and registration was performed in Python 3.6.9 using PlatiPy (www.github.com/pyplati/platipy), which extensively uses SimpleITK [39] and ITK [40]. Our code is available online (www.github.com/rnfinnegan/prostate-biological-model).

### 2.5 Statistical modelling and analysis

The PC biological model comprises a set of three-dimensional, voxel-level statistical models using histology-derived data from the study cohort, mapped to the reference geometry. For each patient the histology slides provided information for only a portion of the entire prostate volume, matching every second *ex vivo* MRI slice and excluded the base and apex, and this incomplete sampling was propagated through the registration process. Therefore, voxels in the reference geometry contained information from a different number of patients. We accounted for this by normalising aggregated data by the sampling frequency at each location.

Annotated tumour lesions were combined to model tumour probability using a normal distribution, a well-known approximation to the empirical binomial distribution of these data. At any location in the reference geometry x, the distribution of the frequency of tumour occurrence, *F*, in a sample of size *n*, was modelled as follows:

(1) F(x)∼B(n(x),p(x))

(2) $$\approx N\left(\mu =n(x)\cdot p(x),\sigma^{2}=n(x)\cdot p(x)\cdot (1-p(x))\right)$$

where *p* is the tumour probability, calculated from empirical data (i.e. the number of samples with a tumour lesion at the location, noting the probabilistic approach to tumour labelling), and $N(\mu ,\sigma^{2})$ denotes a normal distribution with mean μ and variance $\sigma^{2}$. Since the aim was to model the distribution of rates of occurrence, rather than frequency, this distribution was normalised by the sampling frequency. An important characteristic of the tumour probability model is relative variability at each voxel, which was assessed using the coefficient of variation, *CV*, calculated using model parameters in Eq. 1:

(3) $$\mathrm{CV}=\frac{\sigma }{\mu }$$

(4) $$=\frac{\sqrt{\mathrm{np}(1-p)}}{\mathrm{np}}$$

(5) $$=\sqrt{\frac{1-p}{\mathrm{np}}}$$

As expected the relative uncertainty, characterised by the *CV*, increases with lower sampling (*n*). It is also dependent on the empirical rate of tumour occurrence, with higher rates reducing inherent variability.

Individual cell density maps from each patient were also combined to provide voxel-level models. The suitability of two statistical models was evaluated as (1) a normal distribution, and (2) a log-normal distribution, to model the cell density, *C*. These models are defined as follows:

(6) $$C(x)∼N\left(\mu =\frac{1}{n(x)}\overset{n(x)}{\underset{i=1}{\sum }}c_{i}(x),\sigma^{2}=\frac{1}{n(x)}\overset{n(x)}{\underset{i=1}{\sum }}{\left(c_{i}(x)-\mu (x)\right)}^{2}\right)$$

(7) $$\log C(x)∼N\left(\mu =\frac{1}{n(x)}\overset{n(x)}{\underset{i=1}{\sum }}\log c_{i}(x),\sigma^{2}=\frac{1}{n(x)}\overset{n(x)}{\underset{i=1}{\sum }}{\left(\log c_{i}(x)-\mu (x)\right)}^{2}\right)$$

where $c_{i}$ are the individual cell density values and *n* is the number of samples at each location. Relative variability is again presented as the coefficient of variation, however there is no closed form representation as for tumour probability. Using an information theoretical framework, each model’s suitability to approximate the variation in cell density across the study cohort was assessed using the Kullback–Leibler (KL) divergence, $D_{\mathrm{KL}}$. The KL divergence quantified the amount of information lost, in bits, when the normal (or log-normal) model, $C_{\mathrm{model}}$, was used to approximate the empirical data. At each voxel, the Gaussian kernel density estimate (KDE) of the empirical distribution of cell density values, $C_{\mathrm{KDE}}$, was generated with kernel width automatically calculated using Scott’s Rule[41]. The KL divergence was computed as:

(8) $$D_{\mathrm{KL}}(C_{\mathrm{KDE}}\|C_{\mathrm{model}})(x)=\int_{c=0}^{c=\infty }C_{\mathrm{KDE}}(x)(c)\cdot \log_{2}\left(\frac{C_{\mathrm{KDE}}(x)(c)}{C_{\mathrm{model}}(x)(c)}\right)\mathrm{dc}$$

The KDE is a non-parametric representation of the empirical distribution and avoids the need to bin the data using histograms.

## 3 Results

**Fig. 5:**
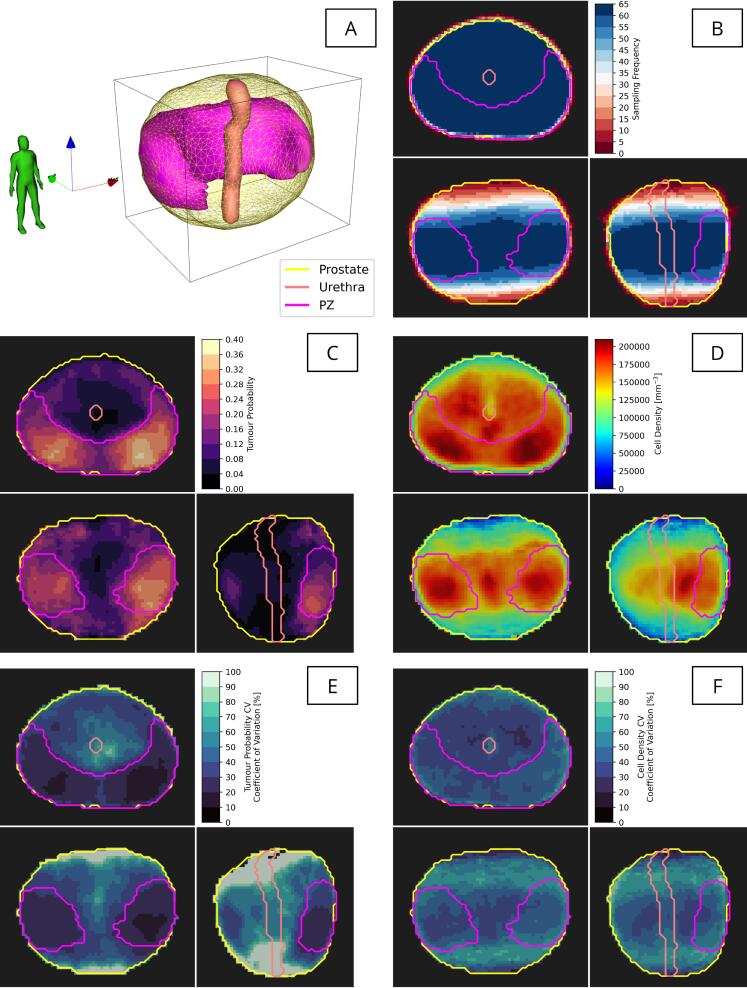
Resulting data comprising the probabilistic prostate cancer biological voxelised model. (A) The reference geometry for the whole prostate, peripheral zone (PZ) and urethra shown in 3D space. (B) Sampling frequency for histology slides, following registration to the reference, (C) tumour probability (any grade), and (D) mean total cell density (shown in orthogonal slices). The variability (coefficient of variation, CV) in tumour probability (E) cell density (F) are presented.

The distance-preserving registration process successfully mapped all individual patient data to the reference geometry. Annotated tumour lesions and cell density maps were aggregated to produce the probabilistic model, illustrated in Fig. 5. As expected, histological sampling frequency was highest in the mid-gland (near 100%) and gradually tapered off towards the prostate apex and base due to the absence of histology data at the extreme base and apex as described earlier. This has implications for the reliability of resulting voxelised statistical models.

**Fig. 6:**
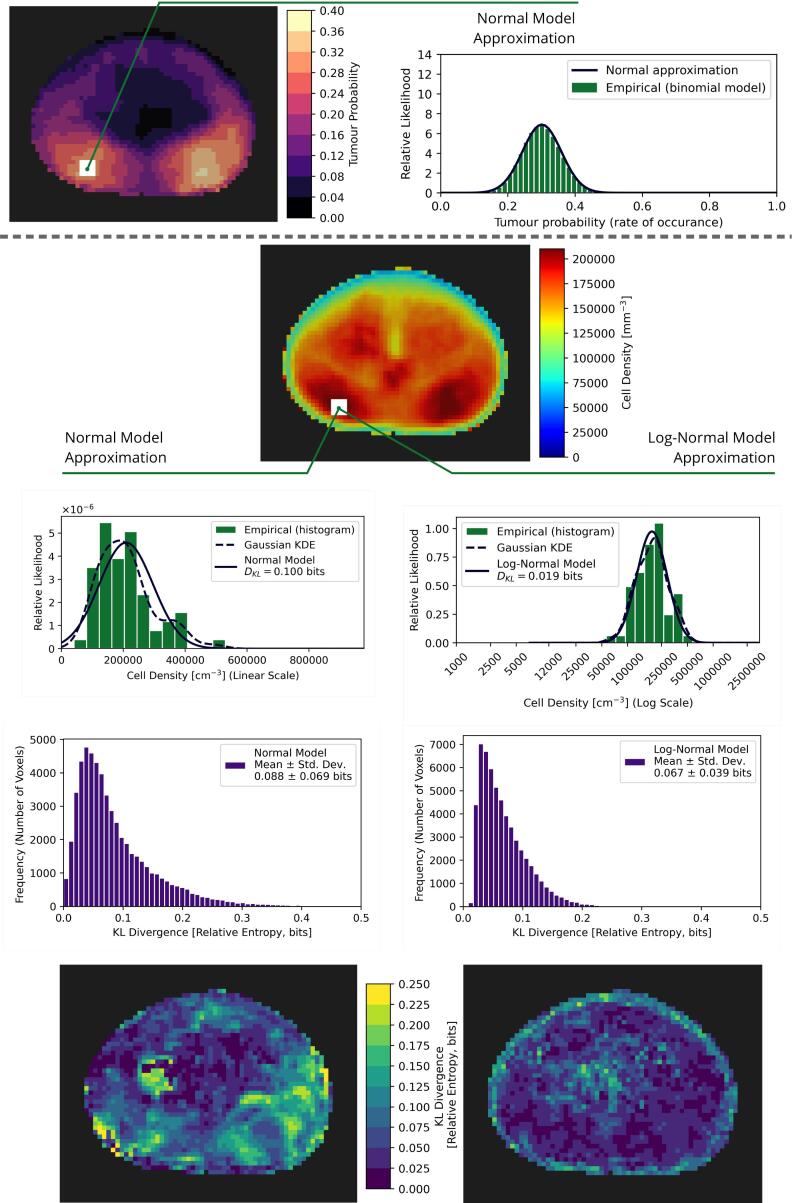
Validation of model selection for the prostate cancer biological voxelised model. Tumour probability was modelled using a normal approximation to the empirical binomial distribution (top). The total cell density (bottom) was modelled using either a normal model (left columns) or a log-normal model (right columns), an example of each model fit to the observations (from the 63 patients) at a single voxel is shown. The Kullback–Leibler (KL) divergence ($D_{\mathrm{KL}}$) was used to quantitatively assess the suitability of each of these models, shown aggregated over all the voxels in a histogram (second to bottom row) and visualised on orthogonal cuts through the reference volume (bottom row).

Voxel-level statistical modelling of tumour probability and cell density is shown in Fig. 6. By construction, the normal model used to approximate the empirical binomial distribution of rates of tumour occurrence accurately encodes variation in tumour probability. For the cell density, the log-normal approximation to the empirical data provided more accurate modelling when compared with the normal approximation, with reduced information loss as measured with the KL divergence. This metric quantified the mean (± std. dev.) relative entropy lost as 0.088±0.069 bits for the normal model and 0.067±0.039 bits for the log-normal model.

## 4 Discussion

In this work, we developed a framework to model biological characteristics of PC and applied it to a study cohort of 63 patients. This required the design and implementation of a new deformable registration process to facilitate accurate and consistent inter-patient registration, and novel voxel-level statistical modelling of tumour probability and cell density to efficiently encode population-based variation.

The design of this framework reflects consideration of inherent uncertainties in the data and processes. For example, interpolation and resampling of the co-registered histology-based data into isotropic voxels was chosen to match the resolution of *in vivo* MRI. We plan to map the model onto individual patient imaging to facilitate biologically-optimised RT research, and here having an model constructed with the same native resolution is optimal. Probabilistic interpolation of tumour probability, another novel contribution, solved the challenge to generate missing annotated histology while efficiently encapsulating tumour grade data. Our new registration process extends existing methods by ensuring deformations preserve relative positions in a pre-defined geometry (constructed using prostate and PZ contours). This was particularly important in this work as it minimised the impact of anatomical variation in the study cohort. Lastly, the reference geometry was constructed using data from all patients, which again represents a design choice to minimise bias.

In this cohort, close to 40% of patients had tumour foci located in the posterolateral regions of the PZ, in agreement with previous reports [42], [43]. The 3D cell density distribution is highly heterogeneous, with maxima coincident with the highest tumour probability and secondary peaks surrounding the urethra. Tumour probability was accurately approximated using a normal model. However, a log-normal model was better suited for approximating cell density, reflecting large variations in cell density between patients, often spanning several orders of magnitude. The KL divergence, mapped in the reference space, highlighted spatial variations in the suitability of either normal or log-normal modelling of cell density. Notably, a log-normal model was comparatively less suitable around the border of the prostate, likely resulting from sampling effects (Fig. 5B), and in the centre of the prostate.

Construction of statistical tumour prevalence models in the prostate has been proposed by several other groups, with a focus on improved biopsy sampling strategies [44], [45]. Early work demonstrated the feasibility of inter-patient histology registration using prostate surface-based deformations combined with internal elastic warping to spatially normalise histological images to a single example image [46], [47]. More recent work of Rusu et al. [28] incorporated patient MRI in the model construction, which enabled analysis of disease appearance, and Rojas et al. [48] demonstrated the potential to generate models specific to clinical parameters, such as prostate-specific antigen (PSA) levels. Further, population-based tumour prevalence maps and *a priori* clinical data can significantly improve the performance of predictive models for tumour delineation [49]. We hypothesise that incorporating biological data, such as cell density, tumour grade, and hypoxia, will improve the performance of AI models used to predict these biological parameters.

This study was subject to several limitations. First, histological slide sampling frequency was much lower at the prostate apex and base, and therefore the model is subject to increased uncertainty in these regions. Second, no ground truth is available for assessment of inter-patient registration, making quantification of uncertainty in the registration process difficult. Third, conversion of cross-sectional to volumetric cell density requires linear scaling: one methodology used previously by our research team [50] computes the scaling factor based on estimated clonogenic cell numbers [51]. Fourth, classification of tumour lesions was performed manually by one experienced urological pathologist and may be subject to observer variability. Emerging techniques in automating histological analysis could provide a more objective process amenable to larger-scale analysis [52], [53], [54]. Finally, a limitation common to all statistical modelling is the finite sample size, in this case comprising 63 complete datasets.

Our previous work has demonstrated the potential for predicting voxel-level PC characteristics from mpMRI, including tumour location [23], cell density [24], and markers of hypoxia [25]. Planned future research will investigate the utility of our model for improving these predictions. Additionally, our model provides a computational testbed for biologically-optimised RT research. Building on our previous work, we will demonstrate the process of co-registration of this model with *in vivo* imaging data to produce biologically optimised dose distributions for external beam RT or brachytherapy applications [55], [56], [57], [58]. Using models for tumour probability and cell density, it is straightforward to derive a map of clonogen density using voxel-wise multiplication [50]. This provides requisite data for radiobiological optimisation of RT dose distributions. Further, variation encoded in our model can be efficiently included from the statistical models of these parameters. Although not explored in this work, the grading of the tumour lesions, and clinical data (PSA levels, age, etc.) could also be incorporated into the model. This provides an opportunity to (1) explore differences in PC between patient sub-populations, (2) investigate the impact these data have on radiobiological models, and (3) personalise the model to individual patients using data acquired prior to RT.

In conclusion, we have developed a novel framework to facilitate the creation of a statistical, voxelised, biological model of PC. This tool encodes 3D spatial information about rates of occurrence and the distribution of cell density in the prostate.

## Declaration of Competing Interest

The authors declare that they have no known competing financial interests or personal relationships that could have appeared to influence the work reported in this paper.
