# Supplementary material for: A statistical, voxelised model of prostate cancer for biologically optimised radiotherapy
Source: Phys Imaging Radiat Oncol. 2022 Mar 6;21:136–45. doi: 10.1016/j.phro.2022.02.011 (PMC8913349; doi:10.1016/j.phro.2022.02.011)
Supplement: Supplementary data 1 [file mmc1.pdf]

# A statistical, voxelised model of prostate cancer for biologically optimised radiotherapy: Supplementary Material

Robert N Finnegan[a,b,c],
Hayley M Reynolds[d],
Martin A Ebert[e,f,g,h],
Yu Sun[a],
Lois Holloway[a,b,c,h,i],
Jonathan R Sykes[a,j,k],
Jason Dowling[a,l,m],
Catherine Mitchell[n],
Scott G Williams[o,p],
Declan G Murphy[o,q], and
Annette Haworth[a]

[a]Institute of Medical Physics, School of Physics, University of Sydney, Sydney, New South Wales, Australia
[b]Liverpool Cancer Therapy Centre, South Western Sydney Local Health District, Liverpool, New South Wales, Australia
[c]Ingham Institute for Applied Medical Research, Liverpool, New South Wales, Australia
[d]Auckland Bioengineering Institute, University of Auckland, New Zealand
[e]Department of Radiation Oncology, Sir Charles Gairdner Hospital, Nedlands, Western Australia, Australia
[f]School of Physics, Mathematics and Computing, University of Western Australia, Crawley, Western Australia, Australia
[g]5D Clinics, Claremont, Western Australia, Australia
[h]Centre for Medical Radiation Physics, University of Wollongong, Wollongong, New South Wales, Australia
[i]South Western Sydney Clinical School, University of New South Wales, Sydney, New South Wales, Australia
[j]Department of Radiation Oncology, Sydney West Radiation Oncology Network, Blacktown Cancer & Haematology Centre, Blacktown, New South Wales, Australia
[k]Department of Radiation Oncology, Sydney West Radiation Oncology Network, Crown Princess Mary Cancer Centre, Westmead, New South Wales, Australia
[l]School of Mathematical and Physical Sciences, University of Newcastle, Newcastle, New South Wales, Australia
[m]CSIRO Health and Biosecurity, The Australian e-Health and Research Centre, Herston, Queensland, Australia
[n]Department of Pathology, Peter MacCallum Cancer Centre, Melbourne, Victoria, Australia
[o]Sir Peter MacCallum Department of Oncology, University of Melbourne, Melbourne, Victoria, Australia
[p]Division of Radiation Oncology and Cancer Imaging, Peter MacCallum Cancer Centre, Melbourne, Victoria, Australia
[q]Division of Cancer Surgery, Peter MacCallum Cancer Centre, Melbourne, Victoria, Australia

March 4, 2022

## Corresponding author and author responsible for statistical analysis

Robert Finnegan
robert.finnegan@sydney.edu.au
A28, Room 604
Physics Rd, University of Sydney
NSW 2006
Australia

## 1 Patient demographic details

Supplementary Table 1: Patient selection details for this study (HREC15/PMCC125).

| Inclusion criteria |
|---|
| Biopsy proven localized prostate cancer with biopsy > 4 weeks prior. |
| Medically suitable for radical prostatectomy and likely to proceed with surgery subject to satisfactory imaging results. |
| Capable of giving informed consent. |
| Age > 18 years. |
| Must be able to communicate in English. |

| Exclusion criteria |
|---|
| Cardiac pacemaker or epicardial wire, cochlear or stapes implant, implanted electronic devices, metal foreign bodies, claustrophobia, sensitivity to Gadolinium contrast agents and weight exceeding gantry specifications for the MRI machine. |
| Inadequate renal function for contrast administration (eGFR <60 mL/min). |
| Any prosthetic implants such as metallic hip prostheses that may cause artifacts in the imaging studies. |
| Previous transurethral resection of prostate (TURP). |
| Prior androgen deprivation therapy. |
| Prior pelvic radiation therapy. |
| Active inflammatory bowel disease. |
| Evidence of active prostatitis. |

Supplementary Table 2: Patient demographic details and clinical information. Pathological T stage is given using AJCC/UICC 8th edition.

| Parameter | | Value |
|---|---|---|
| Age (years) | median (range) | 63 (45 - 74) |
| PSA serum level (ng/mL) | median (range) | 6.7 (2.2 - 42.0) |
| Gleason Score dominant nodule (number of patients) | 3+3 | 4 |
| | 3+4 | 32 |
| | 4+3 | 14 |
| | 4+4 | 2 |
| | 4+5 | 4 |
| | 5+4 | 6 |
| | 5+5 | 1 |
| Pathological T stage | pT2 | 31 |
| | pT3a | 24 |
| | pT3b | 8 |
| Prostate volume ($cm^3$) | median (range) | 30.1 (17.0 - 92.1) |
| Tumour volume ($cm^3$) | median (range) | 2.89 (1.77 - 12.8) |
| Tumour/prostate volume ratio (%) | median (range) | 11.4 (3.25 - 32.3) |

## 2 Ex vivo MRI acquisition details

Each *ex vivo* prostate specimen was scanned using a 3T Siemens Trio Tim machine (Siemens Medical Solutions, Erlangen, Germany) using an 8-channel knee coil. The T2w imaging was acquired with the following parameters:

- turbo spin-echo protocol
- TR/TE = 3670/76 ms
- echo train length = 20
- acquisition matrix = $448 \times 312$
- FOV = $100 \times 69.6$ mm
- slice thickness = 2.5 mm
- Distance factor = 0%
- Concatenations = 1

Additional details can be found in Reynolds et al. [Reynolds2015].

## 3 Details of deep-learning whole prostate segmentation in ex vivo MRI

For the segmentation of the prostate contours, we employed a convolutional neural network with a 3D U-net architecture [cicek2016]. The network was trained to minimise the weighted categorical cross-entropy between predictions and the ground truth. The weights for the cross-entropy were 1 for background and 4 for prostate tissue classes. The dataset of 34 images was split as 31:3 images for the training and testing dataset. During training, the dataset was dynamically augmented changing the brightness, contrast, and randomly flipping the images in the medial-lateral direction.

The optimisation was performed using an ADAM optimiser with a learning rate of $10^{-4}$, momentum parameters of $\beta_1 = 0.9$, and $\beta_2 = 0.999$, with batches of 8 images. The model trained for 2000 epochs without overfitting (the testing loss and accuracy followed a monotonic descending trend). The obtained model has an accuracy of 98.22% in the testing dataset. The accuracy is defined as the percentage of pixels predicted that match the ground truth, i.e., the ratio of true positives and true negatives over all pixels in the dataset.

## 4 Details of the novel deformable registration process with distance-preserving regularisation

The novel deformable registration process with distance-preserving regularisation was used to guide co-registration of patient prostate (and peripheral zone, PZ) contours to the reference geometry.

This began with the definition of the normalised distance, $R$, which is defined at any location $\boldsymbol{x}$, as:

$$R(\boldsymbol{x}) = \frac{d(\boldsymbol{x}, S)}{\max_{\forall \boldsymbol{x}_i \in \mathbb{R}} d(\boldsymbol{x}_i, S)} \tag{1}$$

where $d$ is the Euclidean distance function and $S$ is the structure from which the distance is measured, in this case the prostate (and later, the PZ). This was computed in image space using an established algorithm[Danielsson1980]. Once this map was computed for the reference geometry ($R_{\text{ref.}}$) and patient geometry ($R_{\text{pat.}}$), a multi-resolution algorithm was used for co-registration by optimising the global energy, $E$, by iteratively updating a deformation vector field, $\boldsymbol{s}$, with the update step $\boldsymbol{u}$ in the Lie algebra:

$$E(\boldsymbol{s} + \boldsymbol{u} | R_{\text{ref.}}, R_{\text{pat.}}) = \frac{1}{\sigma_i^2} \| R_{\text{ref.}} - R_{\text{pat.}} \circ (\boldsymbol{s} + \exp(\boldsymbol{u})) \|^2 + \| \nabla \boldsymbol{s} \|^2 \tag{2}$$

$$\boldsymbol{s}_{\text{opt}} = \arg\min_{\boldsymbol{s}} \left( E(\boldsymbol{s} | R_{\text{ref.}}, R_{\text{pat.}}) + E(\boldsymbol{s}^{-1} | R_{\text{pat.}}, R_{\text{ref.}}) \right) \tag{3}$$

where the symmetrisation of the global energy was used to define the optimal deformation vector field, $\boldsymbol{s}_{\text{opt}}$. The second term in this energy term, $\|\nabla \boldsymbol{s}\|^2$, is the square of the gradient magnitude and serves as a regulariser. This term is a measure of the local smoothness of the deformation vector field, and therefore constrains the optimisation to

prevent over-deformation, which often presents as folding and other non-physical deformations. At each optimisation step the optimal deformation field is updated in the log-domain:

$$\boldsymbol{v} = \log \boldsymbol{s} \tag{4}$$

$$\boldsymbol{u} \leftarrow K_{\text{smooth}} \circledast \boldsymbol{u} \tag{5}$$

$$\boldsymbol{v} \leftarrow \boldsymbol{v} + \boldsymbol{u} + \frac{1}{2}[\boldsymbol{v}, \boldsymbol{u}] \tag{6}$$

$$\boldsymbol{s} = \exp \boldsymbol{v} \tag{7}$$

where $[\cdot,\cdot]$ indicates the Lie bracket and $K_{\text{smooth}}$ is the regularisation kernel, defined as a three-dimensional Gaussian function with scale (standard deviation) set to $1.5\times$ voxel size (at each resolution in each stage). Further details of the technical implementation of this approach have been previously reported[Vercauteren2008, Dru2010]. The multi-resolution scheme was run at isotropic image resolutions of 3.2 mm, 1.6 mm, and 0.8 mm (4, 2, and 1 $\times$ the voxel size, respectively).

The diffeomorphic demons registration model is analogous to computing deformation forces inspired by optical flow equations [Thirion1998c]. The symmetric variant, named because the energy term in the cost function is unchanged when reversing the roles of the target and moving images, is known to produce smooth invertible transforms [Vercauteren2009f] which is important to ensure smooth realistic deformation of the prostate and PZ volumes in this work. Regularisation using smoothing of the update field with a Gaussian kernel provides a more computationally efficient method than other approaches, for example using linear elasticity models.
